# GFDP: the gene family database in poplar

**DOI:** 10.1093/database/bay107

**Published:** 2018-10-19

**Authors:** Hao Wang, Hanwei Yan, Huanlong Liu, Rui Liu, Jun Chen, Yan Xiang

**Affiliations:** 1 Laboratory of Modern Biotechnology, School of Forestry and Landscape Architecture, Anhui Agricultural University, Hefei, China; 2 National Engineering Laboratory of Crop Stress Resistance Breeding, School of Life Sciences, Anhui Agricultural University, Hefei, China

## Abstract

A gene family is formed by duplication of a single original gene. Poplar trees (genus Populus) are important, principally because of their ecological and economic benefits, and are one of the most widely distributed and adaptable trees in the world. Systematic identification and annotation of gene family members are primary steps in studying the function and evolution of poplar genomes. Here, we describe the construction of the Gene Family Database in Poplar (GFDP), which contains information that systematically describes 6551 genes distributed in 145 gene families. GFDP is designed to present important biological information, such as gene structure, protein length, isoelectric point and functional and evolutionary information, using highly visual displays. Data and graphs are visualized by a web-based interface. Users can browse and download data through all the major browsers. GFDP provides a comprehensive platform with a solid foundation for further study of poplar gene families. GFDP is free available.

## Introduction

A gene family is made up of multiple genes that are produced by duplication of a single gene during genome evolution. The proteins encoded by duplicated genes share domains (1). Members of the same gene family sometimes form clusters in the genome but more often they are scattered in different locations on the same chromosome or are on different chromosomes. Gene families play important biological roles; for example, transcription factor (TF) families, such as the WRKY (2), heat shock (3), MADS-box (4) and C3H (5) gene families regulate a large number of genes involved in many biological processes. The WRKY TF family plays a significant role in regulating plant development and resistance. The MADS-box TF family is involved in plant signal transduction pathways and development control. The heat shock TF family has significant functions in protecting plants from damage caused by various stresses. Other gene families include the COBRA family, which has significant functions in regulating chromatin and transcription (6), and the IQD family, which plays a critical role in plant cell signaling (7).

Poplar trees (genus *Populus*) possess many excellent characteristics, including economic benefits and as industrial materials, that make them important models for studying genomes and biological functions in plants. *Populus trichocarpa* was the first woody plant to have its genome fully sequenced (8) and the sequences and annotations have been updated in version 3.0 and 3.1. With the availability of poplar genomic data, increasing numbers of gene families have been identified and analyzed. For example, 14-3-3 proteins were found to be involved in signaling pathways to regulate plant development and to protect plants from stresses (9), and the role of the plant-specific TIFY genes in regulating plant stress-responses was reported (10). Many other gene families have been studied in poplar, including the Dof family (11), glutathione S-transferase family (12), the IQD family and the NADPH-cytochrome P450 reductase family (13). Although, many studies on poplar gene families have been reported, no comprehensive database of all the gene families in the poplar genome is currently available.

Here, we describe the construction of the Gene Family Database in Poplar (GFDP) (http://gfdp.ahau-edu.cn/), which contains a total of 145 gene families including 58 TF families. GFDP contains information about gene sequences, including their distribution on the chromosomes, genetic structure and features of the translated protein sequences, as well as functional information, such as motif and domain analysis, gene ontology (GO) annotations, KEGG pathways and PANTHER data. GFDP also contains evolutionary information, including paralogs, orthologs, phylogenetic tree and syntenic analysis. Descriptions of individual gene families and related publications are also provided. GFDP provides a comprehensive web-based search tool with a user-friendly interface to search for detailed annotations and also provides links to external data sets. We expect that GFDP will be a useful platform for researchers interested in the function and evolution of poplar species.

## Materials and methods

### Identification of poplar gene families

The version (v3.0 and v3.1) of the *P. trichocarpa* genome information was obtained from Phytozome (14). Initially, the gene sequences of members of 145 gene families from Arabidopsis were used as query sequences to search the Pfam database to determine conserved domains (http://www.arabidopsis.org/browse/genefamily/index.jsp). Then, the Pfam domain hidden Markov model was applied to the poplar genome sequence to screen for homologous gene sequences. The common domains of the Arabidopsis gene families were used in Blastp (e-value = le−5) to identify corresponding poplar gene families. The identified candidate genes were imported into Pfam (15) and SMART (16) for analysis and inspection, and domain remnant was deleted to ensure the accuracy of the obtained sequence. We named the poplar gene families based on the *Arabidopsis* gene family name. Species-specific genes were identified based on sequences similarity searches against 61 genomes excluding *P. trichocarpa* from phytozome using the BLASTN & BALSTP software (e-value = le−5) (17).

### Annotation of poplar gene families

To obtain more comprehensive information for further analysis, we systematically annotated the candidate genes at both family and gene levels. Each type of data is organized in separate data sets in GFDP.

On each gene family page, a brief introduction, list of family members, their chromosome distribution and bioinformatics analysis information (e.g. motifs, gene structure, phylogenetic tree) are given.

**Figure 1:**
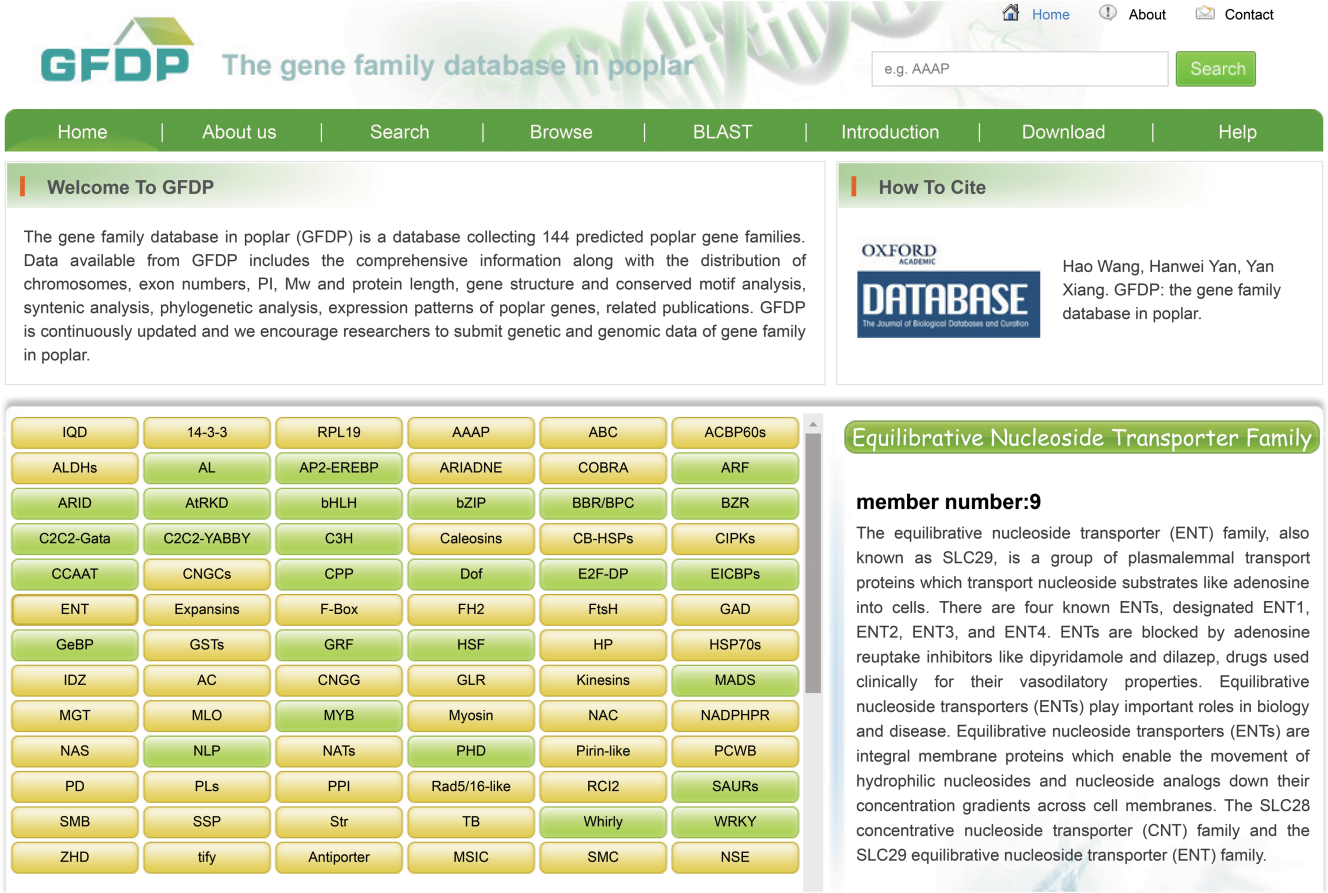
The home page of GFDP.

(i) **Basic gene characteristic** Initially, we obtained basic information about the poplar genes from Phytozome (18), including the nucleotide sequences, protein-coding sequences, protein sequences, chromosome locations, protein lengths and GO annotations. We used the Compute pI/MW tool in ExPasy to obtain the molecular weights and isoelectric points (PIs) of the predicted protein sequences (19).
(ii) **Gene structure and conserved motif analysis** The intron and exon structure for the genes in each family were obtained from the Gene Structure Display Server (20) with the genomic DNA sequences and protein-coding sequences as input. To detect conserved patterns in the poplar gene sequences we used Online Multiple Expectation Maximization for Motif Elicitation (21) with maximum number of sequences set as 15 and optimum motif width between 6 and 200 residues.
(iii) **Phylogenetic analysis** To assess the evolutionary relationship among different members of the same gene family, we used Clustal X (22) to align the multiple amino acid sequences. The result was imported into MEGA 7.0 and a phylogenetic tree was constructed using the neighbor-joining method (23). The phylogenetic tree was tested with the self-development method and bootstrap values obtained in 1000 replicates.
(iv) **Putative homolog annotation** We downloaded the poplar paralogs annotated in Ensembl using Biomart (24). Orthologs across the poplar, Arabidopsis, soybean and grape genomes were also downloaded using Biomart. To ensure the data were accurate, orthologous genes were identified by OrthoMCL with the default parameters described previously (25).
(v) **Expression patterns of poplar genes** The whole-genome microarray data (GSE13990) of nine tissues/organs and developmental stages of *Populus balsamifera* with three biological replicates, which were obtained from NCBI’s GEO database, were used to display the expression patterns of the poplar genes (26). The RNA-seq data was explored among leaf, root and stem xylem transcriptomes under drought, salt or temperature stress (27).
(vi) **Syntenic analysis** To detect the poplar genes that may have resulted from whole-genome duplication events, we used MCscanX software to identify syntenic blocks within the species (28). The syntenic blocks and the genes that they contained were retrieved. An overview of all blocks was visualized using Circos to show the intra-genome syntenic relationships of the poplar genes (29).
(vii) **Comparative statistics** We used the data from each gene in a gene family to show their distribution on chromosomes, exon numbers, isoelectric points, molecular weights and protein lengths.

**Figure 2:**
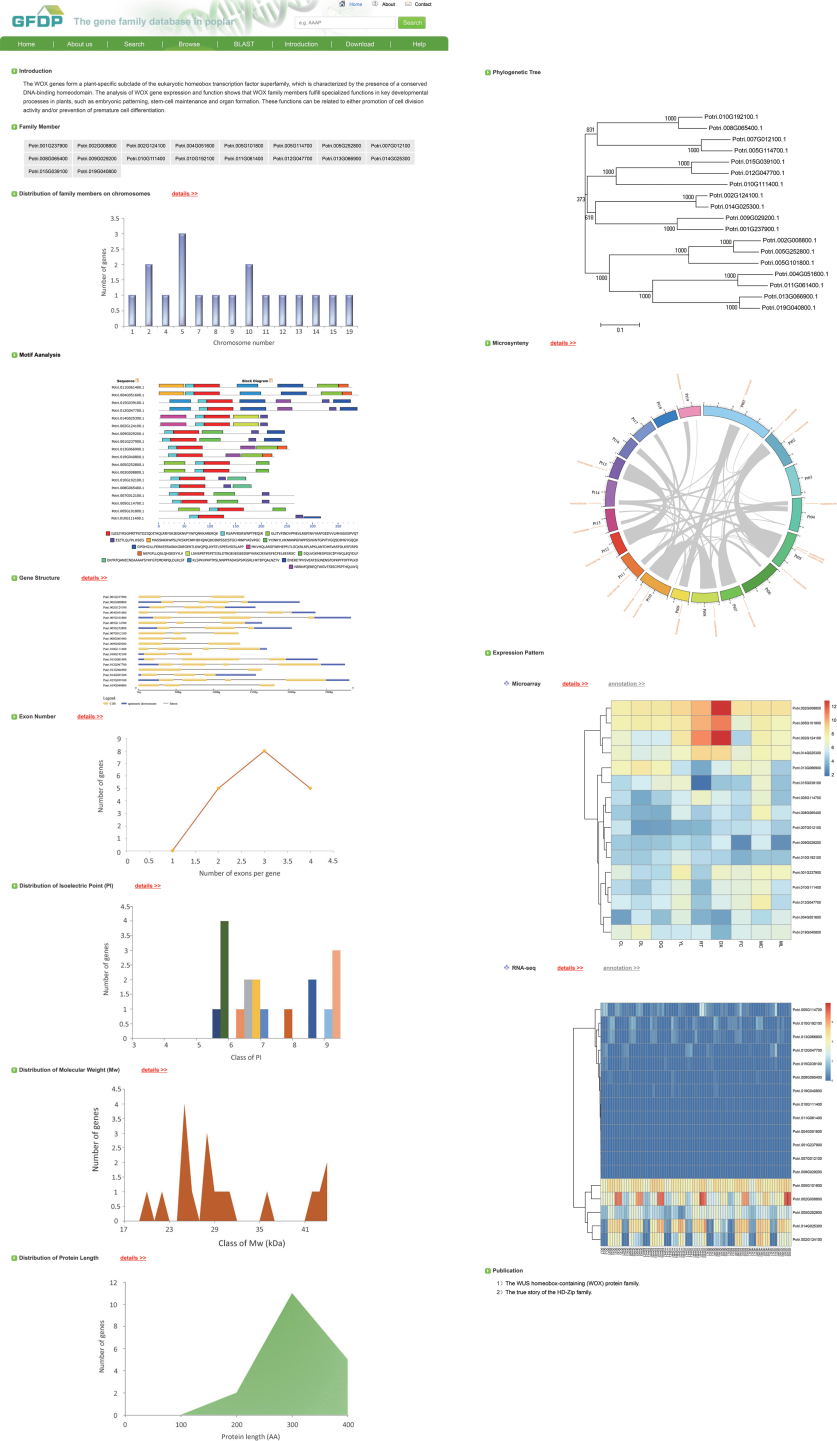
The gene family page of GFDP (e.g. SMC).

**Figure 3:**
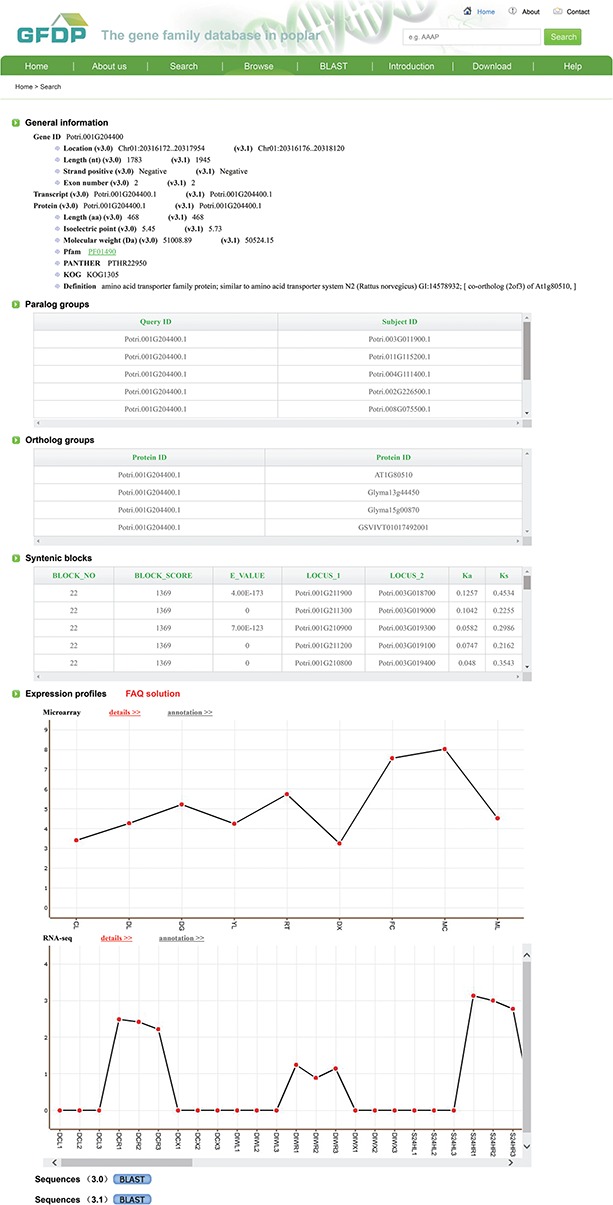
The annotation of a single gene of GFDP (e.g. Potri.001G125000).

### Results

GFDP contains a total of 6551 poplar genes in 145 predicted gene families. The largest family is the Cytochrome P450 family, which contains 393 genes, and the smallest family is the HRT-like family, which contains only one gene.

The GFDP web interface contains eight major sections: Home, About us, Search, Browse, (Basic Local Alignment Search Tool) BLAST, Introduction, Download and Help.

#### Home

The GFDP ‘Home’ page has a brief description of the database and a list of the 145 gene families among which TF families were highlighted in different color. Moving the mouse over a gene family brings up a description of the family and the number of members that it contains (Figure 1).

#### Search

The ‘Search’ function supports simple and advanced searches of the database. For a simple search, users can type a keyword (e.g. a truncated version, the entire ID for a gene family) into the box that appears at the top right of each page. For an advanced search, users can assign the gene ID, gene family name, chromosome, protein length, PI, molecular weight, alias, definition and/or GO term of interest by clicking ‘GO’. The results page displays a list with Gene ID, Gene Family and Chromosome, which can be browsed, and clicking on a gene ID brings up detailed annotations for that gene.

#### Browse

Clicking on ‘Browse’ brings up a list of the 145 gene families separated into three groups—Species-specific genes in poplar, TF gene families and other gene families. Users can click on a family of interest to obtain the detailed annotation information for that family. Each gene family page contains a brief introduction and a list of family members, followed by the analysis results for that family, including distribution of family members on chromosomes, Pfam motifs, gene structure, exon numbers, distribution of PI, distribution of molecular weight, distribution of protein length, phylogenetic tree, microsynteny and gene expression patterns. For some of the gene families, we have provided a list of relevant publications that is linked to external websites where more detailed information can be found (Figure 2). Clicking on a gene in the list of family members displays the detailed annotations for that gene, including gene ID, chromosomal location, strand positive, gene sequence, protein-coding sequence, protein sequence, protein length, chromosome strand, links to GO terms, paralog and ortholog groups, syntenic blocks and expression profiles (Figure 3).

In general, the data in GFDP can be browsed in steps, starting with a gene family from the list, then displaying the annotation information for that gene family, and finally providing the annotations for a single gene.

#### BLAST

The ‘BLAST’ page allows users to enter one or more query sequences in fasta format for BLAST searches against the nucleotide or protein sequences in GFDP. Five BLAST algorithms (blastn, blastp, blastx, tblastn and tblastx) with an E-value ⩽ 1e−5 can be run to find putative homologous poplar sequences.

#### Introduction

The ‘Introduction’ page provides a brief description of each gene family in GFDP and a list of relevant publications to help users find more detailed information about each family.

#### Download

The ‘Download’ page lists all the data that is available for downloading.

#### Help

Clicking on ‘Help’ reveals a pull-down menu from which users can select ‘Methods’ or ‘Links’. Methods displays a page that describes the methods and Links provides links to the data sources and software used to construct GFDP.

## Discussion

The goal of GFDP is to collect information for a comprehensive poplar gene family resource. GFDP contains 145 poplar gene families and 6551 genes with extensive annotations for the members of these families. Among the 145 gene families in GFDP, many gene families have been studied in detail in *Arabidopsis*, but poplar have not. In addition, our GFDP has 773 species-specific genes in poplar. The GFDP interface is user-friendly, which allows users to find information of interest to them easily and effectively. We expect that GFDP will become a valuable resource for the research community, especially for studying the relationships between individual genes and gene families.

GFDP has its own specific features and advantages: (1) Poplar has many outstanding characteristics as a model plant, and poplar gene families have attracted increasing attention in recent years. Gene families have great significance in the study of evolution and gene function. At present, the only database of poplar gene families is DPTF, a database of poplar TFs (30). GFDP contains not only TF gene families, but many other important poplar gene families, such as the 14-3-3, FtsH, NBS-LRR and aldehyde dehydrogenase gene families.

(2) GFDP contains comprehensive genome-wide analysis data of each poplar gene family and each single gene, such as gene expression patterns, phylogenetic tree, syntenic analysis and gene structure. GFDP also provides statistics, such as the gene chromosome distribution, exon numbers, PI, molecular weight and protein length. All these data provide valuable information for further functional and evolutionary research.

(3) GFDP contains information from several public data sources, including Pfam and Expasy, and useful tools, such as NCBI and Phytozome, were used to help annotate the poplar genes and to detect putative homologs. The ‘Link’ option under ‘Help’ allows users to access these useful resources, which can be of great help in understanding more about the gene family information and improves the reliability of the data in GFDP. Importantly, GFDP provides a data submitting system that allows researchers to submit data about a poplar gene family, such as sequences and annotation information. The submitted data are reviewed by the database management administrators before being added to GFDP.

The rapid development of sequencing technologies has led to more and more plant genomes being sequenced making increasing amounts of biological data available to be explored. The availability of GFDP will provide valuable resources to promote the study of poplar genes. We plan to develop new features to further help users query and analyze poplar genes. Our goal is to provide researchers with the largest sharing platform about poplar gene families.

## Conclusions

GFDP is a comprehensive and up-to-date database that we developed focusing on the identification and annotations of poplar gene families in terms of function and evolution. The database provides valuable tools to support research on comparative genomics. GFDP will be updated regularly as more data and information become available. In the future, GFDP will contain more gene annotation and biological data, such as metabolic pathways and co-expression networks. We anticipate that GFDP will serve as an extremely valuable platform for studying poplar genes.

## Declarations

### Availability of data and materials

The GFDP is publicly available and can be accessed at http://gfdp.ahau-edu.cn/.

Operating system(s): Platform independent

Programming languages: PHP, MySQL, HTML and JavaScript

License: Not required

Any restrictions to use by non-academics: None

### Notes

Hao Wang and Hanwei Yan contributed equally to this work.

## Authors' contributions

HW, HY performed integration, constructed the database platform, and wrote the manuscript. HL, JC and RL helped with the design of database platform and update of the database, and provided scientific suggestions and criticisms for improving the manuscript and website. HW, HY and YX participated in the design, helped write the manuscript and supervised the whole project. YX, as the correspondence author, provided financial support for the article. All authors read and approved the final manuscript.

## Authors' information

Laboratory of Modern Biotechnology, School of Forestry and Landscape Architecture, Anhui Agricultural University, Hefei 230036, China; National Engineering Laboratory of Crop Stress Resistance Breeding, School of Life Sciences, Anhui Agricultural University, Hefei 230036, China.

## Consent for publication

Not applicable.

## Ethics approval and consent to participate

Not applicable.

## Supplementary Material

Supplementary Data

## Notes

**Database URL**: http://gfdp.ahau-edu.cn/

## References

[1] Daugherty L.C., Seal R.L., Wright M.W. and Bruford E.A. (2012) Gene family matters: expanding the HGNC resource. Hum. Genomics, 6, 4. 23245209 10.1186/1479-7364-6-4 PMC3437568

[2] He H., Dong Q., Shao Y. et al. (2012) Genome-wide survey and characterization of the WRKY gene family in Populus trichocarpa. Plant Cell Rep., 31, 1199–1217. 22371255 10.1007/s00299-012-1241-0

[3] Fu S., Rogowsky P., Nover L. and Scanlon M.J. (2006) The maize heat shock factor-binding protein paralogs EMP2 and HSBP2 interact non-redundantly with specific heat shock factors. Planta, 224, 42–52. 16331466 10.1007/s00425-005-0191-y

[4] Zhao Y., Li X., Chen W. et al. (2011) Whole-genome survey and characterization of MADS-box gene family in maize and sorghum. Plant Cell Tissue Organ Cult., 105, 159–173.

[5] Chai G., Hu R., Zhang D. et al. (2012) Comprehensive analysis of CCCH zinc finger family in poplar (Populus trichocarpa). BMC Genomics, 13, 253–253. 22708723 10.1186/1471-2164-13-253 PMC3427045

[6] Wu S., Wu M., Dong Q. et al. (2016) Genome-wide identification, classification and expression analysis of the PHD-finger protein family in Populus trichocarpa. Gene, 575, 75. 26314912 10.1016/j.gene.2015.08.042

[7] Ma H., Feng L., Chen Z. et al. (2014) Genome-wide identification and expression analysis of the IQD gene family in Populus trichocarpa. Plant Sci., 229, 96–110. 25443837 10.1016/j.plantsci.2014.08.017

[8] Tuskan G.A., Difazio S., Jansson S. et al. (2006) The Genome of black cottonwood, Populus trichocarpa (Torr. & Gray). Science, 313, 1596. 16973872 10.1126/science.1128691

[9] Tian F., Wang T., Xie Y. et al. (2015) Genome-wide identification, classification, and expression analysis of 14-3-3 gene family in Populus. Plos One, 10, e0123225. 25867623 10.1371/journal.pone.0123225 PMC4395111

[10] Wang Y., Pan F., Chen D. et al. (2017) Genome-wide identification and analysis of the Populus trichocarpa TIFY gene family. Plant Physiol. Biochem., 115, 360–371. 28431355 10.1016/j.plaphy.2017.04.015

[11] Wang H., Zhao S., Gao Y. and Yang J. (2017) Characterization of Dof Transcription Factors and Their Responses to Osmotic Stress in Poplar (Populus trichocarpa). Plos One, 12, e0170210. 28095469 10.1371/journal.pone.0170210 PMC5241002

[12] Lan T., Yang Z.L., Yang X. et al. (2009) Extensive functional diversification of the Populus glutathione S-transferase supergene family. Plant Cell, 21, 3749–3766. 19996377 10.1105/tpc.109.070219 PMC2814494

[13] Ro D.K. and Douglas C.J. (2002) Cloning, Functional Expression, and Subcellular Localization of Multiple NADPH-Cytochrome P450 Reductases from Hybrid Poplar. Plant Physiol., 130, 1837–1851. 12481067 10.1104/pp.008011 PMC166695

[14] Goodstein D.M., Shu S., Russell H. et al. (2012) Phytozome: a comparative platform for green plant genomics. Nucleic Acids Res., 40, D1178–D1186. 22110026 10.1093/nar/gkr944 PMC3245001

[15] Finn R.D., Mistry J., Schusterböckler B. et al. (2006) Pfam: clans, web tools and services. Nucleic Acids Res., 34, D247. 16381856 10.1093/nar/gkj149 PMC1347511

[16] Letunic I., Copley R.R., Schmidt S. et al. (2004) SMART 4.0: towards genomic data integration. Nucleic Acids Res., 32, D142. 14681379 10.1093/nar/gkh088 PMC308822

[17] Yang L., Zou M., Fu B. and He S. (2013) Genome-wide identification, characterization, and expression analysis of lineage-specific genes within zebrafish. BMC Genomics, 14, 65–65. 23368736 10.1186/1471-2164-14-65 PMC3599513

[18] Rhee S.Y., Beavis W., Berardini T.Z. et al. (2003) The Arabidopsis Information Resource (TAIR): a model organism database providing a centralized, curated gateway to Arabidopsis biology, research materials and community. Nucleic Acids Res., 31, 224. 12519987 10.1093/nar/gkg076 PMC165523

[19] Artimo P., Jonnalagedda M., Arnold K. et al. (2012) ExPASy: SIB bioinformatics resource portal. Nucleic Acids Res., 40, W597. 22661580 10.1093/nar/gks400 PMC3394269

[20] Guo A.Y., Zhu Q.H., Chen X. et al. (2007) GSDS: a gene structure display server. Hereditas, 29, 1023. 17681935

[21] Bailey T.L. and Elkan C. (1995) The value of prior knowledge in discovering motifs with MEME. pp. 21–29. 7584439

[22] Thompson J.D., Gibson T.J., Plewniak F. et al. (1997) The CLUSTAL_X windows interface: flexible strategies for multiple sequence alignment aided by quality analysis tools. Nucleic Acids Res., 25, 4876–4882. 9396791 10.1093/nar/25.24.4876 PMC147148

[23] Kumar S., Stecher G. and Tamura K. (2016) MEGA7: Molecular Evolutionary Genetics Analysis Version 7.0 for Bigger Datasets. Mol. Biol. Evol., 33, 1870. 27004904 10.1093/molbev/msw054 PMC8210823

[24] Herrero J., Muffato M., Beal K. et al. (2016) Ensembl comparative genomics resources. *Database (Oxford)*, 2016, bav096. 26896847 10.1093/database/bav096 PMC4761110

[25] Li L., Stoeckert C.J. and Roos D.S. (2003) OrthoMCL: Identification of Ortholog Groups for Eukaryotic Genomes. Genome Res., 13, 2178. 12952885 10.1101/gr.1224503 PMC403725

[26] Wilkins O., Nahal H., Foong J. et al. (2009) Expansion and diversification of the Populus R2R3-MYB family of transcription factors. Plant Physiol., 149, 981–993. 19091872 10.1104/pp.108.132795 PMC2633813

[27] Filichkin S.A., Hamilton M., Dharmawardhana P.D. et al. (2018) Abiotic Stresses Modulate Landscape of Poplar Transcriptome via Alternative Splicing, Differential Intron Retention, and Isoform Ratio Switching. Front. Plant Sci., 9:5. 10.3389/fpls.2018.00005 PMC5816337 29483921

[28] Wang Y., Tang H., Debarry J.D. et al. (2012) MCScanX: A toolkit for detection and evolutionary analysis of gene synteny and collinearity. Nucleic Acids Res., 40, e49–e49. 22217600 10.1093/nar/gkr1293 PMC3326336

[29] Krzywinski M., Schein J., Birol İ. et al. (2009) Circos: an information aesthetic for comparative genomics. Genome Res., 19, 1639–1645. 19541911 10.1101/gr.092759.109 PMC2752132

[30] Zhu Q.H., Guo A.Y., Gao G. et al. (2007) DPTF: a database of poplar transcription factors. Bioinformatics, 23, 1307–1308. 17392330 10.1093/bioinformatics/btm113

